# Supplementary material for: Age-dependent assessment of genes involved in cellular senescence, telomere and mitochondrial pathways in human lung tissue of smokers, COPD and IPF: Associations with SARS-CoV-2 COVID-19 ACE2-TMPRSS2-Furin-DPP4 axis
Source: Res Sq. 2020 Jun 15:rs.3.rs-35347. Preprint. [Version 1] doi: 10.21203/rs.3.rs-35347/v1 (PMC7336702; doi:10.21203/rs.3.rs-35347/v1)

<u>**Supplementary File**</u>

Age-dependent assessment of genes involved in cellular senescence, telomere and mitochondrial pathways in human lung tissue of smokers, COPD and IPF: Associations with SARS-CoV-2 COVID-19 ACE2-TMPRSS2-Furin-DPP4 axis

Krishna P. Maremanda[1], Isaac K. Sundar[1], Dongmei Li[2] and Irfan Rahman[1*]

[1]Department of Environmental Medicine, [2] Department of Clinical & Translational Research,
University of Rochester Medical Center,
Rochester, NY, USA

Supplementary figure 1.

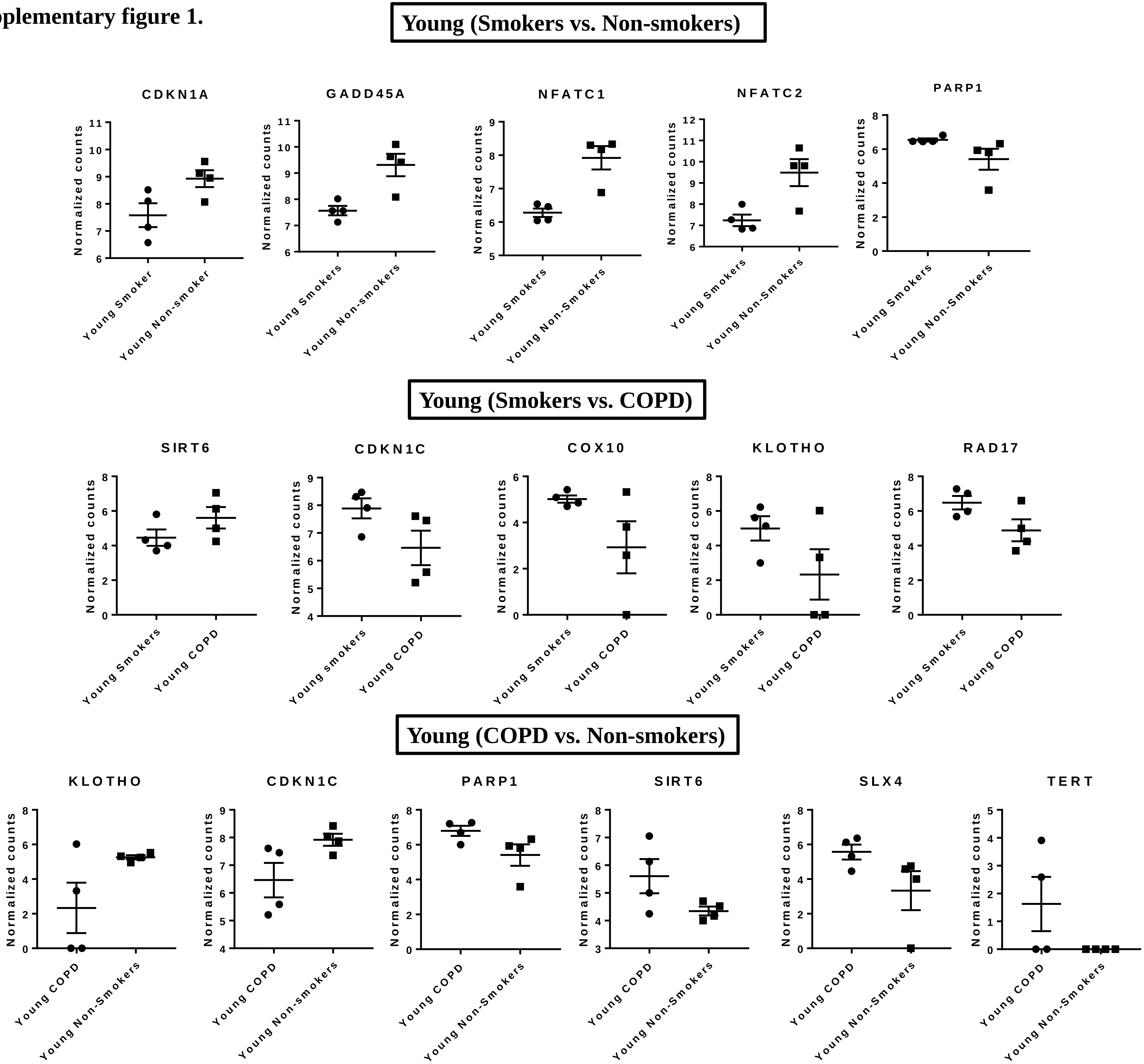

Supplementary figure 2.

Old (Smokers vs. Non-smokers)

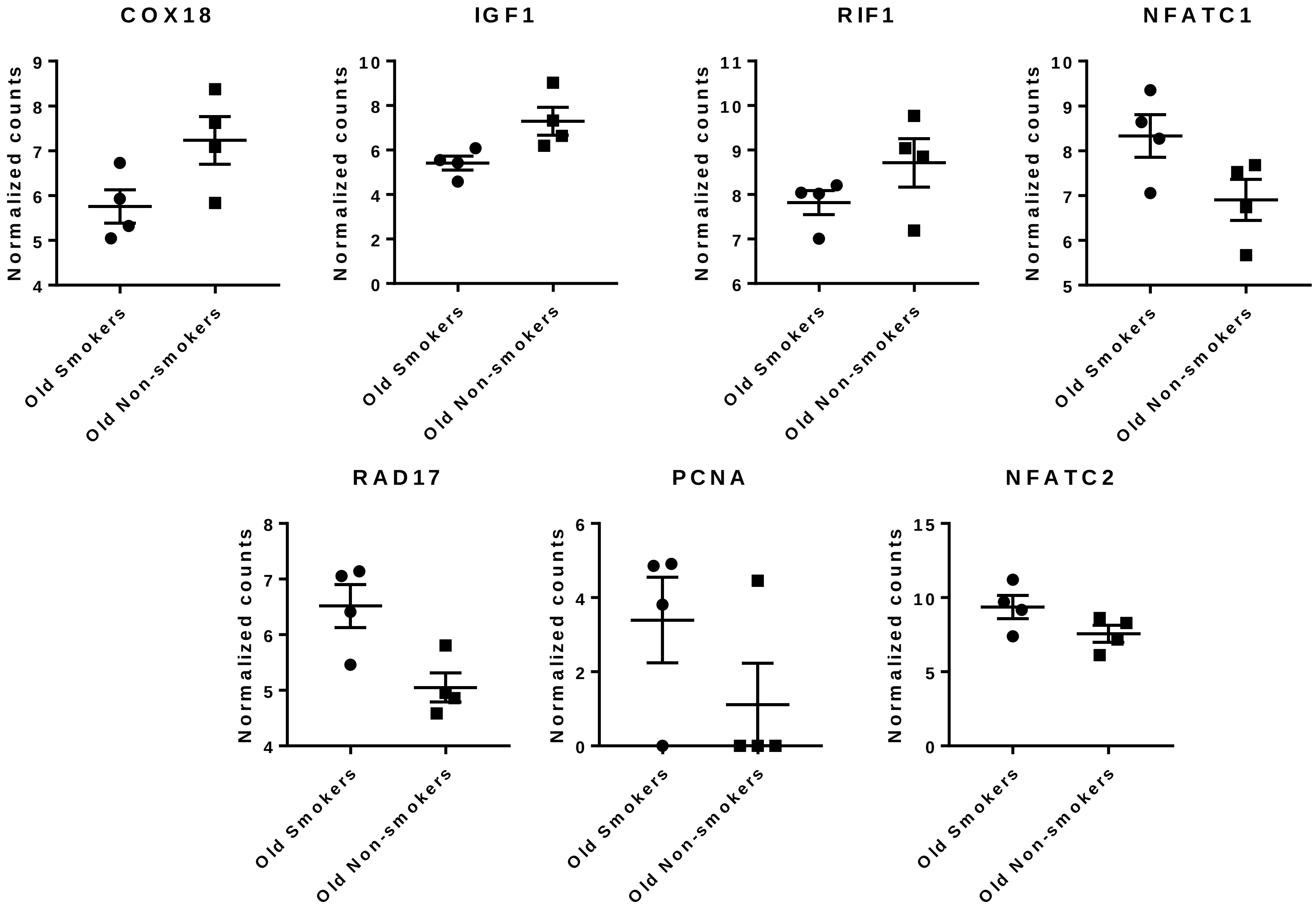

Supplementary figure 3.

Old (Smokers vs. COPD)

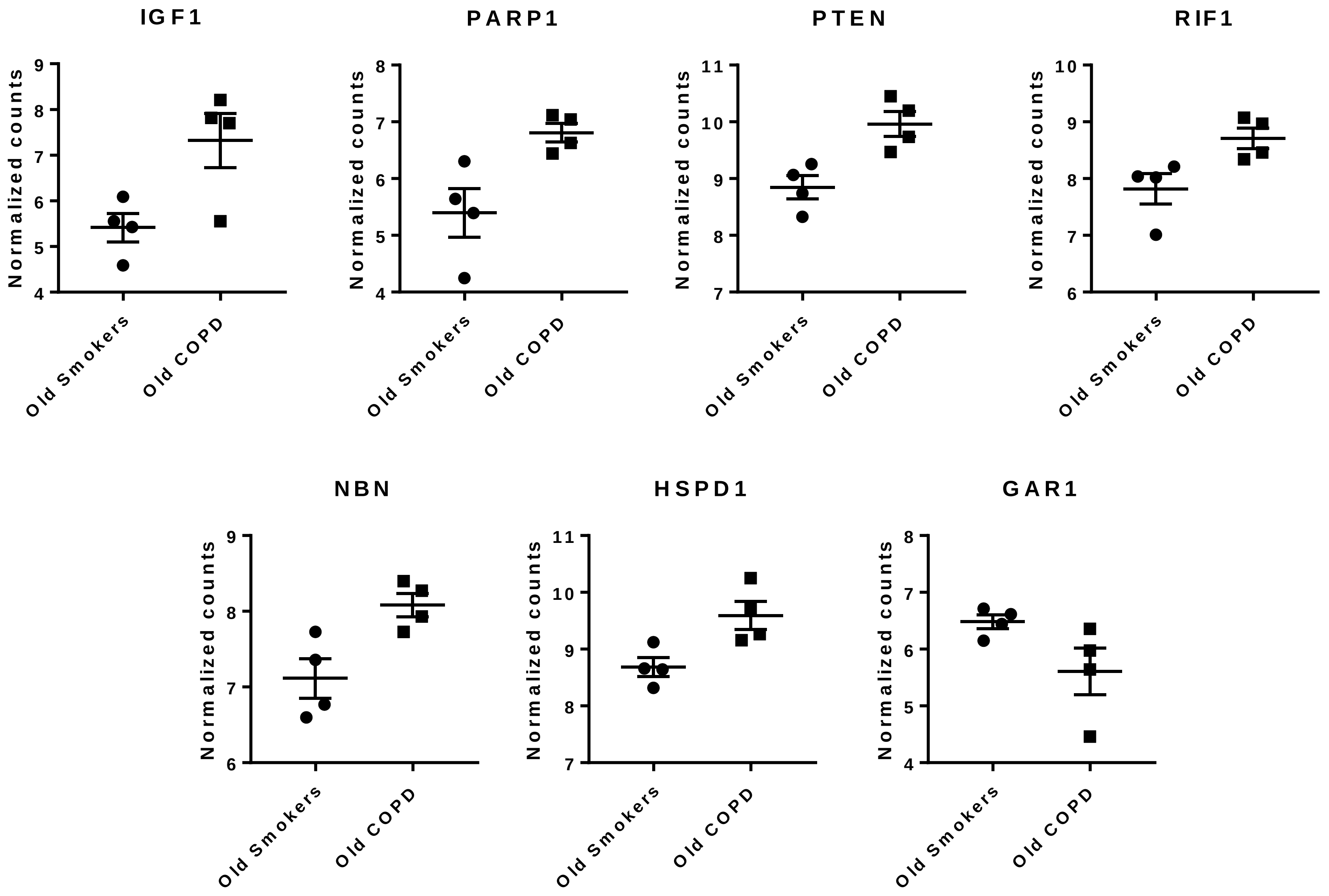

Old (COPD vs. Non-smokers)

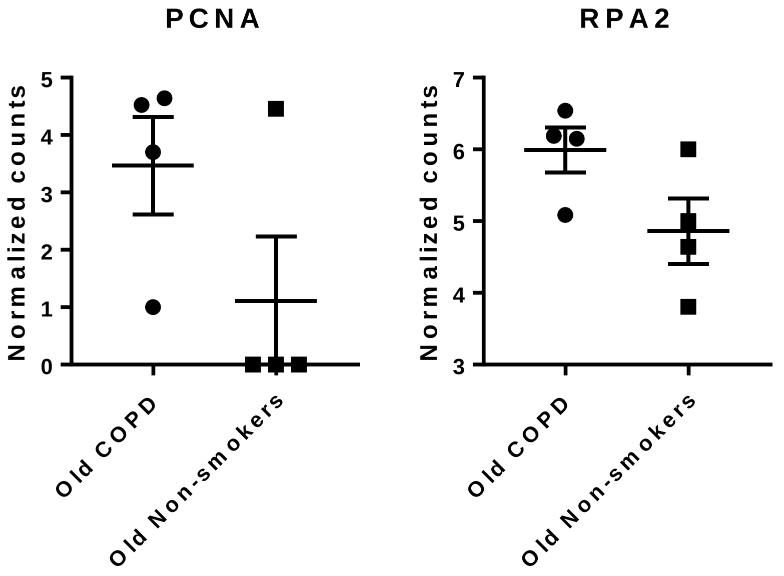

Supplementary figure 4.

Young Non-smokers vs. Old Non-smokers

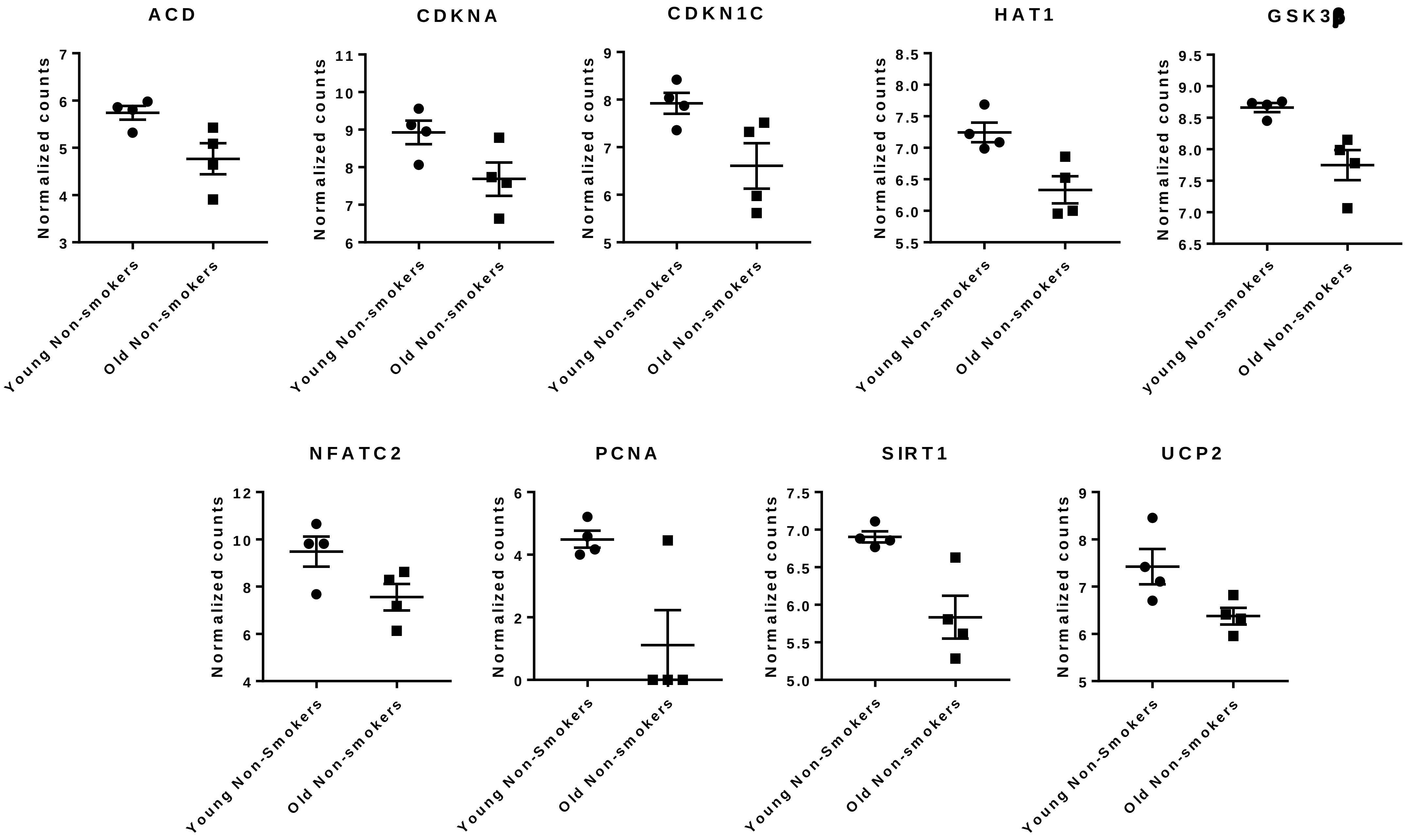

Supplementary figure 5.

Young Smokers vs. Old Smokers

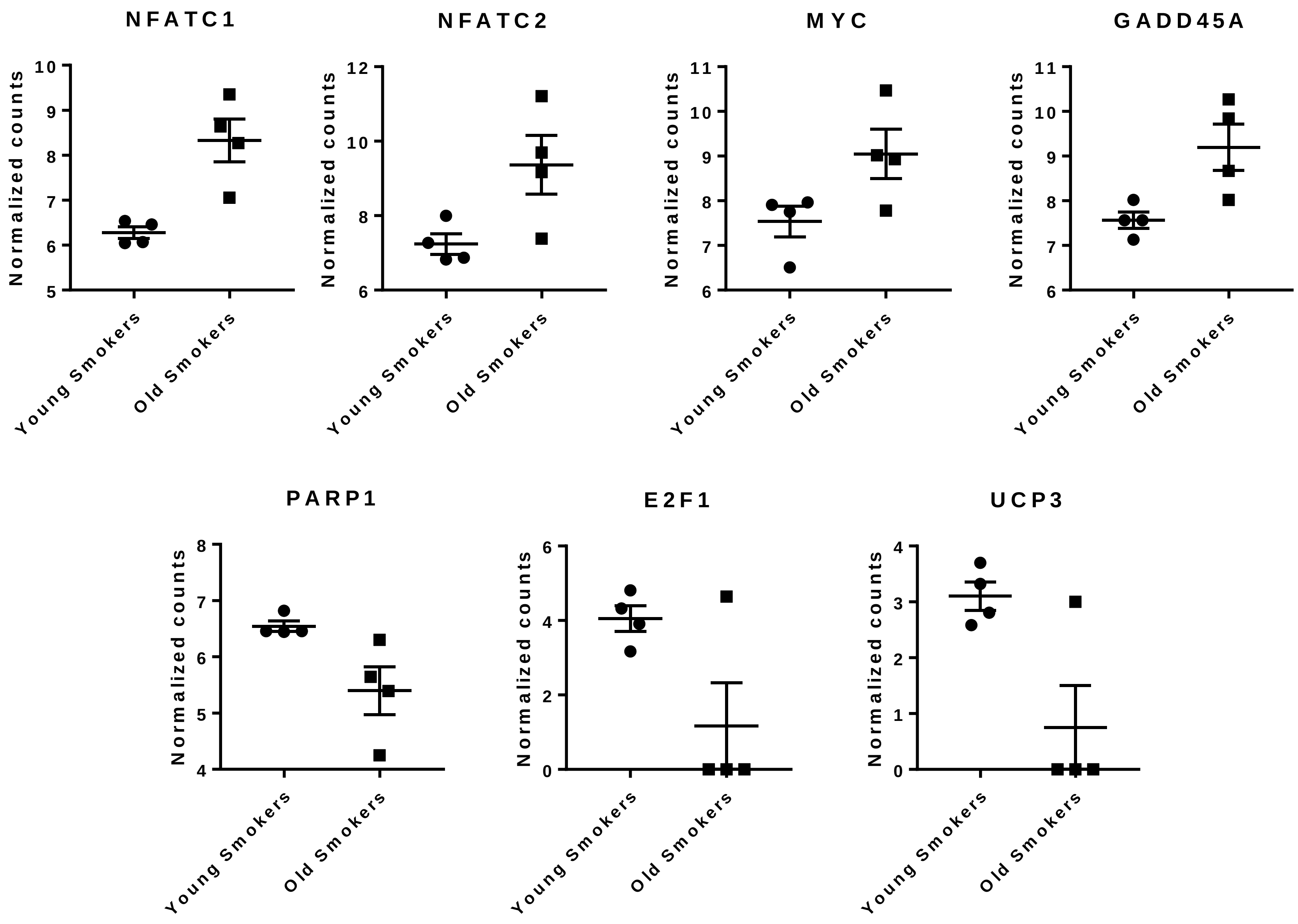

Supplementary figure 6.

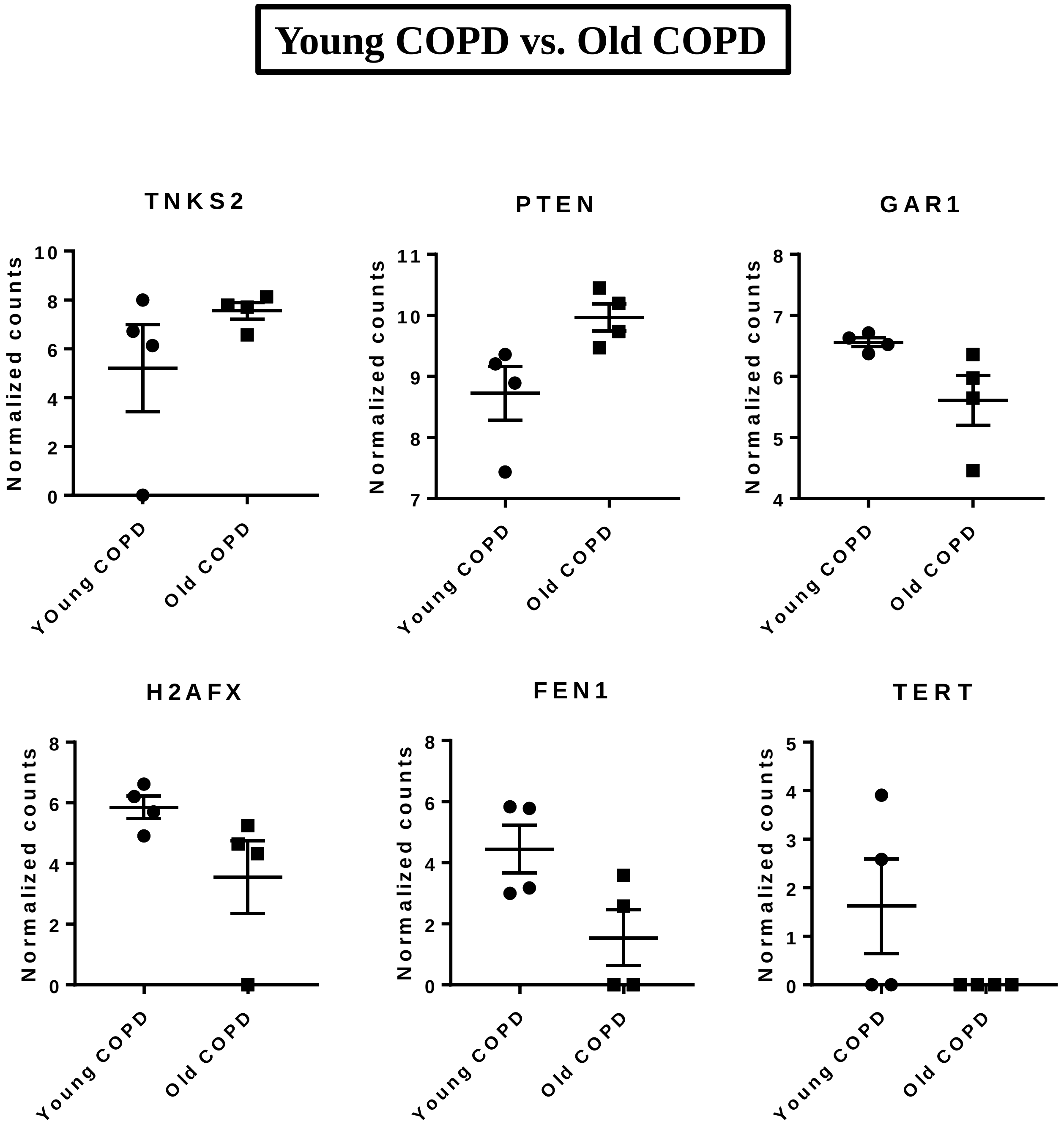

Supplementary figure 7.

Smokers vs. Non-smokers

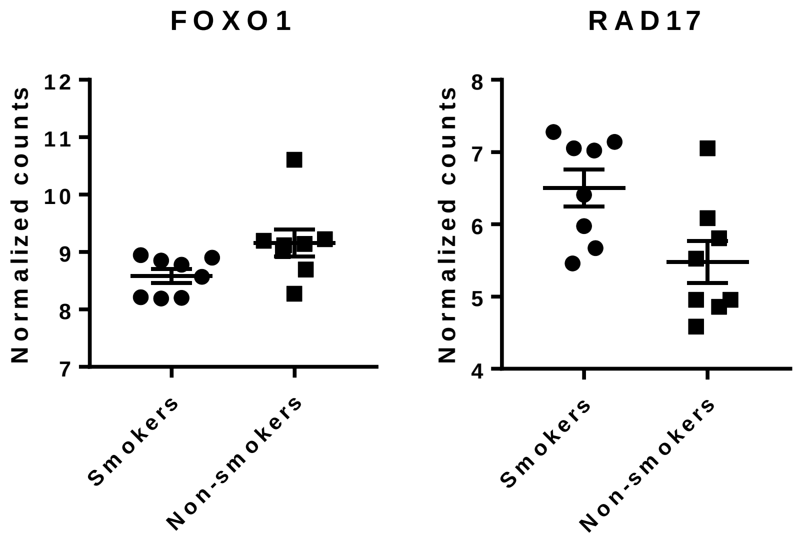

Smokers vs. COPD

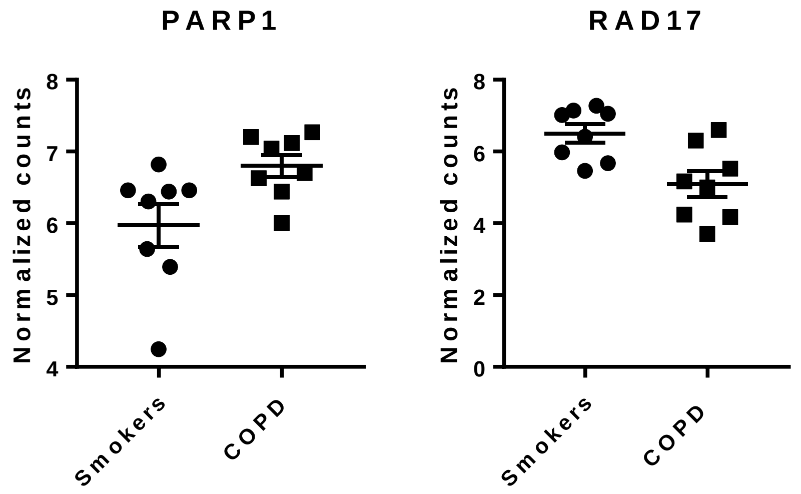

COPD vs. Non-smokers

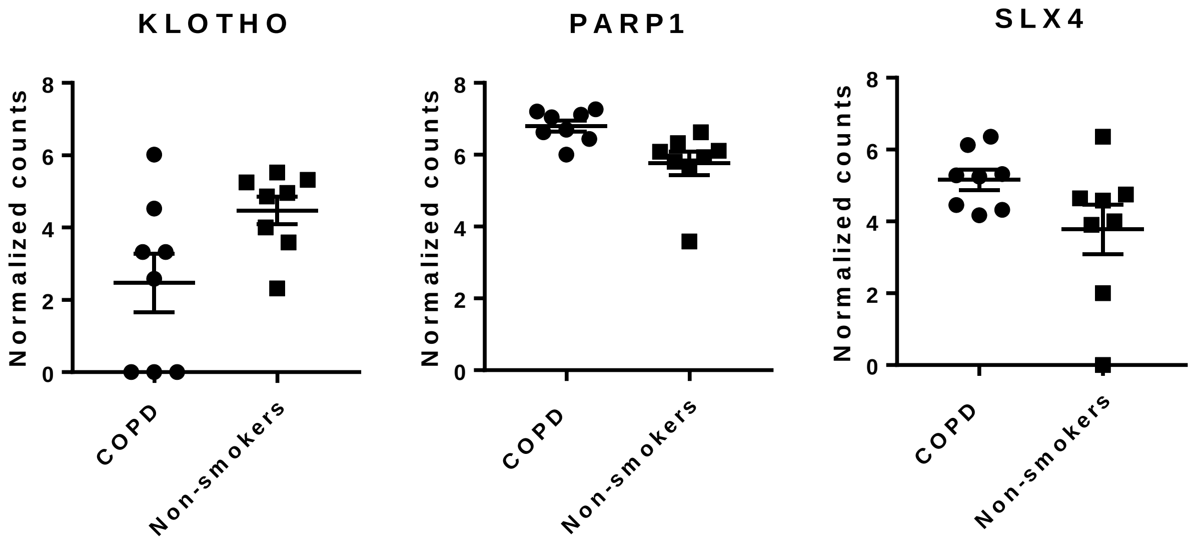

Supplementary figure 8.

Young (COPD vs. IPF)

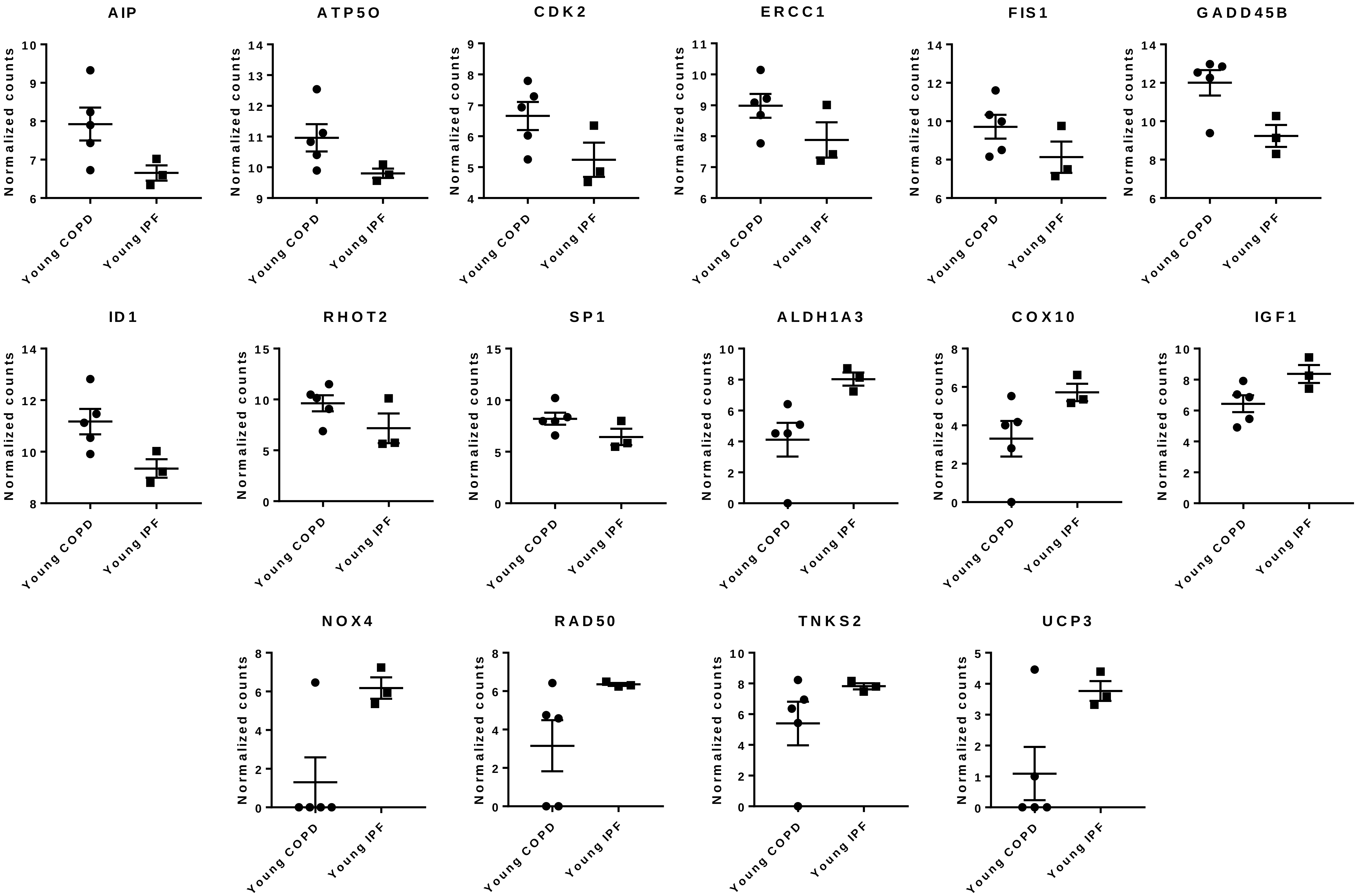

Supplementary figure 9.

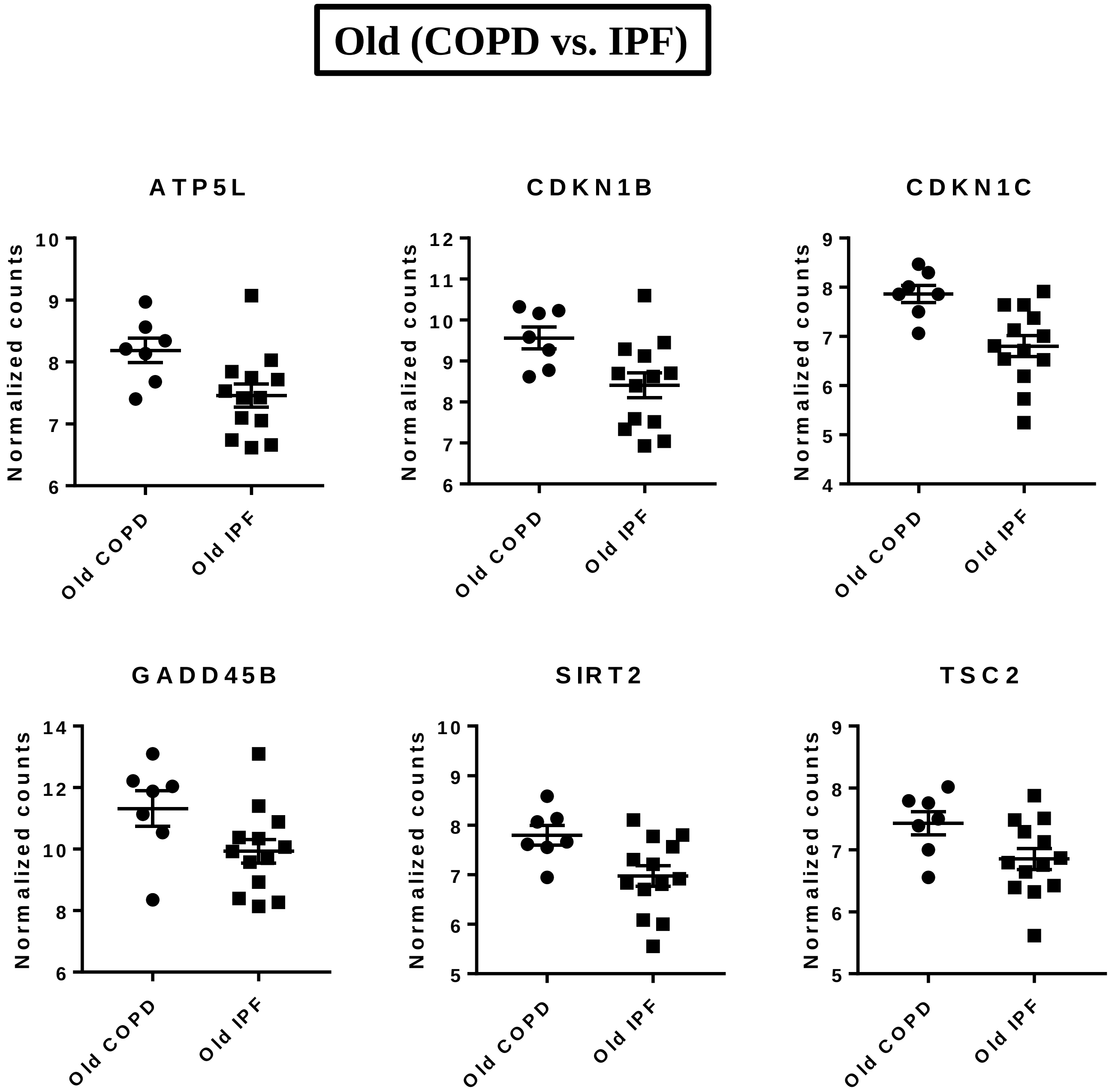

Supplementary Figure 10. Full unedited gels/blots for Fig. 10 (original and unprocessed)

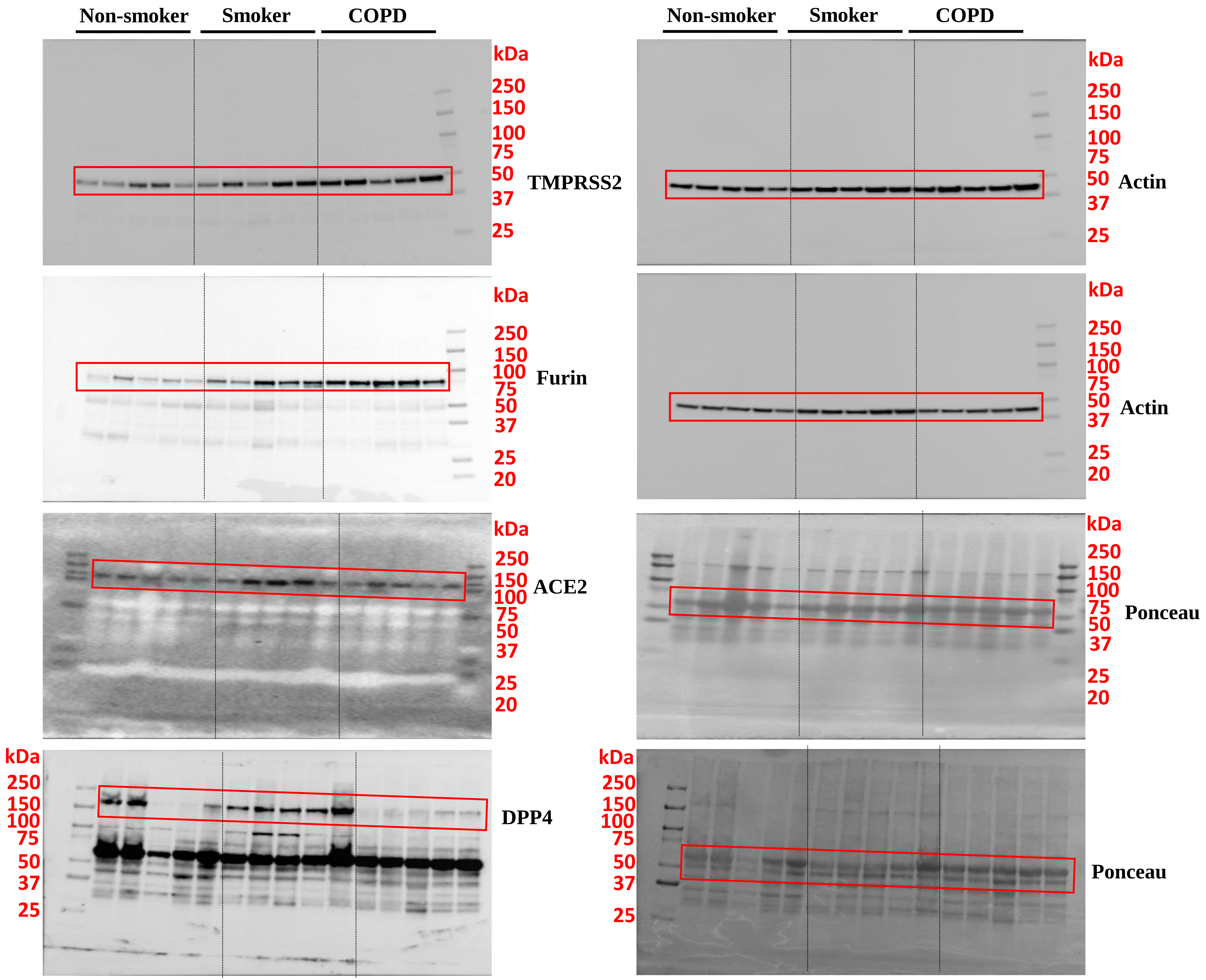

Supplementary Figure 11.

Full unedited gels/blots for Fig. 11 (original and unprocessed)

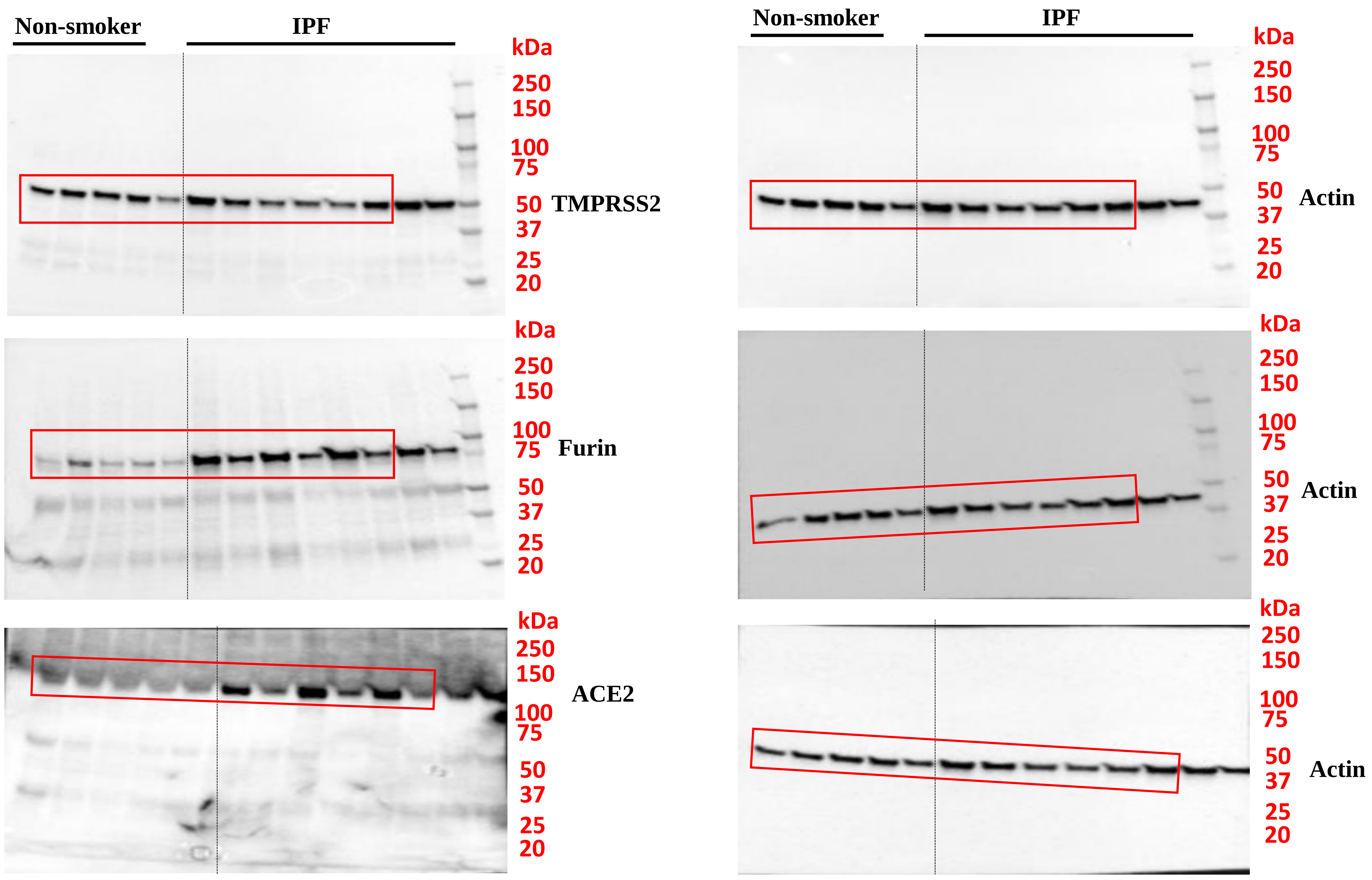

Supplement: Supplement [file SupplementaryFigures111ir.pdf]
